# Expression of Forkhead box P3 isoforms in hepatocellular carcinoma cells

**DOI:** 10.1186/2051-1426-2-S3-P197

**Published:** 2014-11-06

**Authors:** George Chen, Jianwei Ren, Rocky Ho, Paul Lai

**Affiliations:** 1 Dept of Surgery, Prince of Wales Hospital, The Chinese University of Hong Kong, Hong Kong

## 

Forkhead box P3 (FOXP3) is well known as a key regulator of immune homeostasis. The abnormal expression of FOXP3 can contribute to features of several cancers including metastasis and invasion, and it has also been associated with prognosis of some cancers. However, little information is available for the expression of FOXP3 in hepatocellular carcinoma (HCC). In this study, we analyzed the expression of FOXP3 in HCC cells and tissue samples. Using RT-PCR, we found that HCC cells could not only express the full-length Foxp3 (Foxp3FL) but also two alternative-splicing variant forms lacking the exon 2 (Foxp3E2) or exon 7 (Foxp3E7). The expression of Foxp3FL, Foxp3E2 and Foxp3E7 was confirmed by sequencing. In order to examine the functions of Foxp3FL, Foxp3E2 and Foxp3E7, we cloned these 3 forms of Foxp3 into pcDNA3.1, forming pcDNAFoxp3FL, pcDNAFoxp3E2 and pcDNAFoxp3E7. Overexpression of these 3 forms of Foxp3 led to obvious reduction of HCC cell proliferation compared with non-transfected cells or cells transfected with the empty vector. We found that the inhibitory effect of Foxp3FL was the strongest whereas the effect of Foxp3E2 was the weakest. It is concluded that HCC can express Foxp3FL together with two variant isoforms, Foxp3E2 and Foxp3E7 and that all these 3 isoforms are functional in term of their ability to inhibit the tumor cell proliferation.

